# Comment on Carson et al: Strengthening global health security – lessons learned from Public Health England’s International Health Regulations strengthening project

**DOI:** 10.1186/s12992-022-00844-2

**Published:** 2022-05-15

**Authors:** Ahmed Razavi, Samuel Collins, Anne Wilson

**Affiliations:** Global Operations, Health Protection Operations, UK Health Security Agency (formally Public Health England), London, UK

Dear Editor,

We thank the Itad team for their evaluation of the Department of Health and Social Care’s (DHSC) Public Health England^1^ (PHE) International Health Regulations (IHR) Strengthening project, with the summary of their evaluation findings published in your journal [1].

The Itad team identified a number of areas where we have made significant progress at the mid-point of our project cycle. Itad’s evaluation demonstrated how we have built up strong relationships with partner countries and are highly valued by stakeholders. At the time of submission of this paper the IHR team have constructively taken on board a number of ITADs recommendations and continue to work on the others, implementing a number of measures to further improve the Project.

Greater financial transparency with transfer of greater financial responsibility to in-country teams, supported by appropriate competencies within teams, and supportive governance and oversight arrangements, will be in place for the next project cycle. However, there are organisational corporate controls that place limitations on the extent to which financial management can be devolved to in-country teams. The project has also invested a lot of time in restructuring monitoring and evaluation to take on board Itad’s recommendations and have a new Theory of Change and logframe indicators to be able to better evidence the impact of the work we do. Assessing the quality of the inputs we provide is a key tenet of this.

The IHR team will also continue to identify complementarities both within the UK Government (e.g. Fleming Fund, DEFRA, FCDO) and with other partner organisations in order to capitalise on opportunities for synergy and to work collaboratively. Global Health Security is a growing priority area for the UK government and we continue to work with our colleagues from the Foreign, Commonwealth and Development Office (FCDO), Fleming Fund and UK-Public Health Rapid Support team to ensure we are aligned. One Health is a key example of this, with discussions ongoing across UK government departments on potential collaborative work.

The Project is already expanding the composition of in-country teams to include combinations of resident PHE staff and locally recruited staff who will work alongside UK-based technical staff. Virtual delivery, by reducing international travel, when applied in combination with face to face engagement simultaneously offers a method to continue activities despite the impact of COVID-19 and an opportunity for enhanced Value for Money. With additional staff capacity the IHR Project team now have more robust mechanisms in place to ensure evidence of data quality is obtained, with designated M&E champions within all country teams. As the project matures from the scoping phase to a programme phase, it continues to improve, adapt and refine our processes to better support strengthening global health security.

As a new approach to PHE’s global health work, the IHR Project has been ‘building the ship while sailing it’. The positive assessment of our performance is an indication of the future potential of the project to achieve its goals and we look forward to the endline evaluation by Itad which we anticipate will demonstrate improvements we have made and our implementation of recommendations from the midterm evaluation.

Yours Sincerely,

The IHR Strengthening Project team.

## Data Availability

Not applicable.

## Footnotes

1 Public Health England transitioned in to the UK Health Security Agency (UKHSA) in October 2021
